# Gene Therapies in Atrial Fibrillation

**DOI:** 10.1007/s12265-025-10685-0

**Published:** 2025-10-14

**Authors:** Cian O’Donnell, Aleksei Mikhailov, Shin Yoo, Asish Ghosh, Rishi Arora

**Affiliations:** Section of Cardiology, Department of Medicine, University of Chicago, Chicago, IL USA

**Keywords:** Atrial fibrillation, Gene therapy, Structural remodeling, Electrical remodeling, Oxidative stress

## Abstract

Atrial fibrillation (AF) is the most common sustained cardiac arrhythmia, yet current therapies—including drugs and catheter ablation—remain suboptimal. Gene therapy offers a promising way to modulate AF’s molecular drivers. This review summarizes recent preclinical studies using viral and non-viral vectors, atrial-specific delivery strategies, and key targets such as ion channels, fibrosis, and oxidative stress. Despite promising results, no AF gene therapy has FDA approval, due to challenges in atrial targeting, immune control, and durable expression. Closing this translational gap is critical for future AF gene therapy.

**Graphical Abstract:**

Gene therapy for atrial fibrillation leverages viral and non-viral vectors to address targets including fibrosis, ion channels, and oxidative stress.

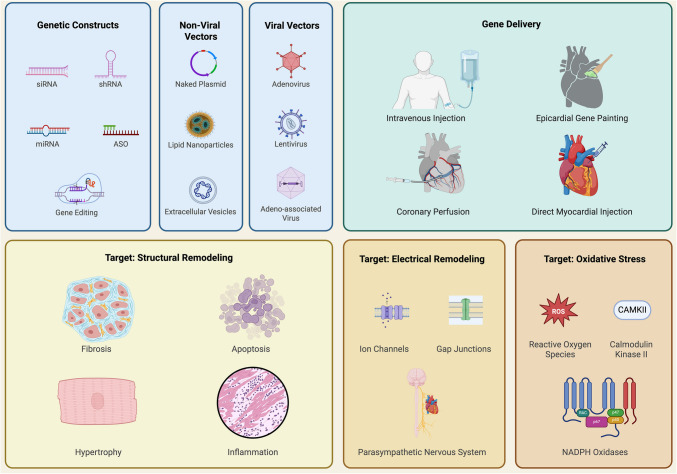

## Introduction

Atrial fibrillation (AF) affects an estimated 59.7 million people globally, a number projected to rise with increasing lifespan and comorbidities such as obesity [1, 2]. Despite advances in ablation and pharmacological therapies, recurrence rates remain high and treatments targeting AF’s molecular mechanisms are still lacking.

Although cardiovascular gene therapy trials are expanding, few focus specifically on AF. Developing AF-targeted therapies is complicated by its multifactorial etiology—including diet, lifestyle, and genetic predispositions [3]. However, a more complete understanding of the molecular mechanisms underlying AF is rapidly developing.

This review outlines gene-based strategies for modulating AF mechanisms, summarizes delivery methods to the fibrillating atria, and highlights targetable structural, electrical, and oxidative pathways. We conclude with a discussion of key opportunities and challenges in advancing AF gene therapy to the clinic.

## Myocardial Gene Transfer

A fundamental step in AF gene therapy is delivering genetic material into the myocardium to alter the disease’s molecular mechanisms. No universally accepted delivery method exists. Instead, success requires balancing vector selection, promoter specificity, and minimizing immunogenicity and toxicity. Researchers must also ensure compatibility between genetic constructs and vectors, while achieving efficient atrial cell uptake.

### Genetic Constructs

The genetic material delivered to the atrial myocardium must be controlled by a promoter region upstream of the transgene. Cardiac-specific promoters such as cardiac troponin T (cTNT) and alpha myosin heavy chain (αMHC), and atrial-specific promoters like atrial natriuretic peptide (ANP) have shown promise [4–6]. In one study, systemic injection of adeno-associated virus (AAV) carrying the ANP promoter drove robust, dose-dependent GFP expression restricted to atria, with no detectable signal in ventricles or extracardiac tissues at doses up to 5 × 10^12^ genome copies per mouse [4].

Small interfering RNAs (siRNAs) are short, double-stranded non-coding RNAs that bind to complementary mRNA sequences, promoting their degradation via the RNA-induced silencing complex (RISC), thereby suppressing gene expression [7]. A recent preclinical study used siRNA to suppress a gene involved in cardiac fibroblast proliferation and activation. Specifically, siRNA targeting STK38L reduced expression of pro-fibrotic markers POSTN and COL1A1 by 30% and significantly inhibited fibroblast proliferation and migration in transforming growth factor-β1 (TGF-β1) stimulated mouse cardiac fibroblasts [8].

Short Hairpin RNA (shRNA) operates using the RNA interference (RNAi) pathway but is transcribed intracellularly from DNA vectors in a stem-loop structure, enabling stable and long-term gene silencing and reduced off-target effects [9]. In a recent study, shRNA-mediated knockdown of bone morphogenetic protein 2 (BMP2) in neonatal rat atrial fibroblasts upregulated the Pyrin Domain-Containing Protein 3 (NLRP3) inflammasome and secretion of IL-1β and IL-6. Since BMP2 suppresses fibroblast activation and restrains NLRP3-driven inflammation, its inhibition promoted a profibrotic, proinflammatory phenotype, exacerbating molecular processes that drive atrial fibrosis and AF pathogenesis [10].

MicroRNAs (miRNAs) are naturally occurring non-coding RNAs that regulate gene expression by binding to the 3’ untranslated region of mRNAs. Targeted miRNA regulation has shown promise in AF preclinical studies, including reductions in fibrosis and pathological electrical remodeling [11]. For example, inhibiting miR-21—an miRNA associated with enhanced fibrosis in AF—using anti-miR-21 in cultured human atrial fibroblasts exposed to media from tachypaced HL-1 atrial cardiomyocytes significantly reduced markers of activated fibroblasts, including α-smooth muscle actin and connective tissue growth factor [12].

siRNA offers potent yet transient knockdown and is simpler to manufacture, while vector-delivered shRNA provides stronger, longer-lasting gene suppression suited for chronic interventions. miRNA-based approaches, although broader in regulatory effect, are typically milder and risk greater off‑target modulation compared to the more targeted RNAi strategies [9, 13].

Another mechanism by which genes may be silenced post-transcriptionally are antisense oligonucleotides (ASOs). ASOs can selectively target specific RNA sequences and affect protein translation by binding to complementary mRNA sequences, leading to degradation via RNase H, steric blocking of translation, or modulation of splicing to alter protein expression [14]. In a rat model, ASO-mediated inhibition of SK3 ion channels downregulated SK3 protein expression by 48%, duration of AF episodes in response to burst pacing was reduced by 78%, and the number of spontaneous AF episodes were decreased by 68% [15].

Gene editing tools offer the opportunity to directly modify genes associated with AF. Early preclinical studies are using CRISPR/Cas9 to understand the genetic components of AF, including the effect of transcriptional regulators on electrical remodeling and AF susceptibility [16, 17]. Newer technologies like base editing and prime editing offer greater precision and fewer off-target effects, making them increasingly attractive options [18].

### Non-Viral Vectors

Naked plasmids are circular DNA constructs that can carry larger transgenes than viral vectors and do not elicit immune responses [19]. Their low uptake can be improved by electroporation, which opens cell membrane pores with electrical pulses. This method increased atrial uptake of naked plasmids by 15–20-fold in an animal AF model [20, 21].

Lipid nanoparticles (LNPs) are biocompatible, scalable vectors for delivering genetic material [22]. The LNP-based therapy Patisiran, approved for amyloid transthyretin amyloidosis, highlights their therapeutic potential [23]. In AF, mRNA-loaded LNPs may target fibrosis and other pathways [24]. In a hypertensive mouse model, CD5-targeted LNPs encoding fibroblast activation protein-targeted chimeric antigen receptor (CAR) mRNA led to the in vivo generation of transient CAR T cells, which normalized cardiac function including left ventricular end diastolic and systolic volumes and ejection fraction. Optimized ionizable LNPs achieved over 80% in vitro transfection and doubled heart tissue expression in vivo [25].

Extracellular vesicles such as exosomes can transport genetic materials with low immunogenicity [26]. Exosomes have been studied in cardiac regeneration [27]. To date, scientists have studied atria and epicardial fat-derived exosome contribution to fibrosis [28–30]. Adipose-derived mesenchymal stem cell exosomes enriched in miR-320d were shown to inhibit apoptosis and enhance cell viability in cardiomyocytes exposed to rapid electrical stimulation by downregulating STAT3 [31].

Polymers, lipids, and nanomaterials are also being studied for cardiac gene delivery [32, 33].

### Viral Vectors

Viral vectors, which exploit the natural ability of viruses to deliver genetic material to host cells, are widely used in gene therapy research [34].

Adenovirus (Ad) is a double-stranded DNA virus with 30–50% cardiac transduction efficiency in vivo [35]. In a porcine model, Ad reduced postoperative AF risk [36]. Ad gene expression is transient, typically lasting two weeks. In New Zealand White rabbits, epicardial delivery of Ad-KCNH2-G628S resulted in atrial expression lasting up to 42 days without adverse effects on cardiac function, serum IL-6, or ECG parameters, confirming prolonged and safe adenoviral gene expression [37].

AAVs, derived from adenovirus, are now the most widely used vectors in clinical gene therapy, achieving cardiac transduction efficiencies of 80% and higher [38, 39]. Capsid engineering is enabling alterations in viral tropism for specific cell types [40]. Despite early success in non-cardiac uses, AAVs have caused fatal liver and systemic toxicity [41]. An early preclinical application of AAV9 in AF involved gene replacement of myosin light chain 4 (*Myl4)* in a homozygous loss-of-function rat model. Intravenous AAV9 delivery of wild-type *Myl4* in neonatal rats restored atrial MYL4 protein levels, rescued electrocardiographic abnormalities including P-wave absence, significantly improved left atrial dilation (reducing diastolic diameter from 6.62 mm to 5.48 mm), restored left ventricular ejection fraction (LVEF), reduced inflammatory cytokines, and attenuated atrial fibrosis [42].

Lentiviral vectors (LVs) are single-stranded RNA retroviruses that exhibit stable gene expression, a moderate carrying capacity, and in vivo transduction efficiencies generally ranging from 20–40% [34, 43]. While LVs can transduce post-mitotic cardiomyocytes, they integrate into the host genome, posing a risk of insertional mutagenesis [34]. The risk of insertional mutagenesis has prevented the use of LV-based therapies in clinical trials. However, preclinical research into LV-based therapies is ongoing. In one study, LVs were used to overexpress a microRNA that is downregulated in AF, successfully restoring cardiac function and reducing atrial remodeling in rats [44]. In another study, lentiviral vectors were used to deliver shRNA targeting the long non-coding RNA myocardial infarction-associated transcript directly into the atria of rats, resulting in a significant increase in the atrial effective refractory period (AERP), reduction of AF duration, and a marked decrease in the expression of myocardial fibrosis markers including collagen I, collagen III, CTGF, and TGF-β1 [45].

## Myocardial Gene Delivery

Intravenous (IV) administration is achieved by injecting the gene therapy directly into the bloodstream. While IV represents a minimally invasive delivery technique it also distributes the therapy systemically and is typically absorbed by non-target organs. For example, AAV therapies can accumulate in the liver, causing toxicity and potentially leading to off-target effects [46].

Cardiac perfusion uses the coronary vasculature to deliver gene therapies locally while avoiding invasive surgical access. Delivery is typically via antegrade coronary artery or retrograde coronary sinus injection, sometimes in combination [47]. The technique involves occluding the vessel and allowing the therapy to dwell and perfuse adjacent tissue. Antegrade injection has been used for cardiac gene therapy but is limited by rapid washout and ischemia risk, whereas coronary sinus occlusion improves transduction rates [48, 49]. Mechanical circulatory support may further enhance efficacy [50]. Retrograde coronary sinus injection enables longer, safer occlusion, and has shown success for ventricular gene transfer, but atrial delivery has not yet been systematically compared between antegrade and retrograde routes [51].

Epicardial gene painting is a surgical approach in which a gene transfer vector is applied directly to the epicardial surface of the heart [52]. This technique leverages a poloxamer/trypsin compound to prolong vector dwelling time and transmural penetration at the target site [52]. An advantage of this approach is that the gene therapy can be applied directly to the heart, thus minimizing off-target effects. A limitation of this technique is the need for surgical access to the epicardial surface. In AF, epicardial gene painting has shown efficacy in multiple animal studies [37, 53, 54]. Epicardial gene painting of adenoviral vectors encoding connexins (Cx40 or Cx43) in a porcine model significantly improved conduction velocity, reduced atrial fibrillation inducibility, and increased sinus rhythm probability (by 2–fourfold) compared to untreated animals after one week of rapid atrial pacing [54].

Direct myocardial injection optimizes localized delivery of gene therapy by injecting it directly into the target site while minimizing off-target effects [49]. Direct myocardial injection has been successfully applied in preclinical studies of AF, targeting diverse mechanisms including oxidative stress and fibrosis [20, 55, 56]. Challenges of this approach include the need for direct access to the heart, atrial wall thickness, and the limited diffusion of the injected therapy, which remains near the injection site, making pan-atrial delivery prohibitively inefficient in a clinical setting.

## Gene Therapy Targets for Atrial Fibrillation

AF involves electrical and structural remodeling of the atrial myocardium, forming reentry circuits and sustaining arrhythmia. Advances in understanding its molecular pathways have enabled targeted gene therapy strategies aimed at structural, electrical, and oxidative mechanisms.

### Structural Remodeling

Fibrosis is a well-established contributor to AF pathogenesis [57]. A key mediator of fibrosis is TGF-β signaling. Through a network of different pathways, TGF-β1 signaling promotes collagen deposition in the atrial myocardium [58]. A gene therapy approach targeting TGF-β1 signaling in AF significantly reduced fibrosis in a canine model; specifically, plasmid-mediated expression of a dominant-negative TGF-β type II receptor in the posterior left atrium decreased fibrosis by 50%, reduced AF inducibility, lowered conduction heterogeneity, and reduced the maximum slope of the action potential restitution curve (from 3.10 ± 0.78 to 1.09 ± 0.17) compared to controls [55].

Apoptosis regulation is a promising AF gene therapy strategy. In canine models, dysregulated superoxide dismutase-1 (SOD1), which manages oxidative stress, contributes to AF progression. Lentiviral inhibition of microRNA 206, a SOD1 regulator, in the left ganglionated plexus reduced AF susceptibility and prolonged action potential duration [59]. Inhibiting caspase-3, a key apoptotic enzyme, offers another strategy to reduce AF-related apoptosis. In a porcine model, adenoviral siRNA targeting caspase-3 reduced atrial apoptosis and delayed persistent AF onset [60].

Inflammation contributes to both the establishment and progression of the AF substrate [61]. The NLRP3 inflammasome plays a significant role in AF development mediated by pro-inflammatory cytokines and electrical remodeling [62]. AAV9-mediated cardiomyocyte-specific knockdown of NLRP3 significantly reduced AF inducibility to 20% after AAV9-shNLRP3 treatment vs 77.8% in scramble treated controls and significantly attenuated structural atrial remodeling, demonstrating the therapeutic potential of AAV-based gene knockdown of inflammasome components in AF [63].

### Electrical Remodeling

Potassium currents (IK) play a crucial role in atrial repolarization, and their dysregulation contributes significantly to the development and maintenance of AF. In AF, increased IK1 and IKACh currents shorten the action potential duration (APD) and effective refractory period (ERP), facilitating reentry circuits and arrhythmogenesis [64]. NOX2-induced oxidative stress has been shown to activate protein kinase C epsilon (PKCε), which in turn enhances the acetylcholine-regulated potassium current (IKH)—a contributor to atrial electrical remodeling and atrial fibrillation (AF) vulnerability. This maladaptive pathway was attenuated by shRNA-mediated NOX2 knockdown, which reduced PKCε translocation and IKH upregulation [20].

Late sodium currents (INa,L) contribute to AF by prolonging the action potential duration and increasing intracellular calcium overload, which promotes electrical instability and triggered activity through delayed afterdepolarizations (DADs) [65]. Traditional therapy strategies targeting late sodium currents focus on reducing NaV1.5 channel activity or modulating its regulators, such as calmodulin kinase II (CaMKII), to prevent excessive sodium influx [66]. Although gene therapy approaches have targeted CaMKII in other arrhythmia contexts, no current gene therapy strategy has yet been shown to directly suppress INa,L in atrial fibrillation models. The role of CaMKII in AF pathophysiology and its potential as a gene therapy target is addressed in the ‘Oxidative Stress’ section of this review.

L-type calcium channels (LTCC) facilitate inward calcium current (ICA,L), which is integral to the cardiac action potential, and their reduction has been implicated in AF [67]. Gene therapy approaches that increase (ICA,L) density through upregulation or gene replacement could reverse AF pathophysiology. However, a potential downside to this approach is intracellular calcium toxicity, which is characteristic of early AF and may itself drive the subsequent downregulation of L-type calcium channels. Indeed, atrial myocytes from chronic AF patients exhibit reduced (ICA,L) density compared to sinus-rhythm controls [68]. Notably, in a rabbit AF model, lentiviral knockdown of autophagy gene ATG7 restored Cav1.2 expression and normalized (ICA,L), prolonged AERP, and reduced AF vulnerability, demonstrating a promising gene-based strategy to rescue calcium currents without directly overexpressing ion channels [69].

Gap junctions play a central role in electrical conduction in the heart and dysregulated gap junctions are implicated in AF [70]. Gap junctions are composed of connexins, with Cx40 and Cx43 being predominant in atrial tissue [70]. In a porcine burst atrial pacing model, epicardial gene injection plus electroporation of adenoviral vectors encoding Cx43 caused a 2.5-fold increase in Cx43 protein, preserved LVEF, and enhanced conduction velocity compared to controls. Moreover, sustained AF did not develop in any treated pigs (0/7) compared to 5/6 in controls [71].

The parasympathetic nervous system plays a role in AF pathophysiology primarily through vagal nerve signaling. Acetylcholine release from vagal terminals activates muscarinic type-2 receptors (M2Rs) in atrial myocytes, inhibiting adenylate cyclase and activating IK-ACh, which shortens atrial refractoriness and promotes reentrant arrhythmias [72]. Targeted inhibition of G-protein signaling in the posterior left atrium has been explored as a gene therapy approach to selectively attenuate parasympathetic influences and reduce AF inducibility. In a canine model, adenoviral delivery of the inhibitory G-protein α-subunit (Gαi) inhibitor Gαi2ct to the posterior left atrium significantly increased the effective refractory period and reduced AF inducibility by 69% [73].

### Oxidative Stress

Accumulation of reactive oxygen species (ROS) through overproduction or insufficient antioxidant enzymes leads to oxidative stress, which is a major contributor to AF pathogenesis [74]. Oxidative stress contributes to electrical remodeling by causing ion channel dysfunction and structural remodeling by driving inflammation and fibrosis [75–77]. Oxidative stress also causes calcium handling impairment, and autonomic nervous system dysfunction [78, 79]. Targeting sources of oxidative stress has emerged as a potential strategy for AF gene therapy.

Under normal conditions, the NADPH Oxidase (NOX) family of enzymes, in particular NOX2 and NOX4, maintains physiologic levels of ROS in the heart, supporting functions such as cell signaling and hypertrophy [80]. The core enzymatic reaction catalyzed by NOX involves the transfer of electrons from NADPH to oxygen, resulting in the formation of superoxide [80]. An example of an AF trigger that upregulates NOX enzymes includes atrial stretch [81]. As previously detailed, attenuation of NOX2 expression reduced oxidative injury and prevented AF onset in a canine model [20].

CaMKII regulates cardiac excitation–contraction coupling, translating calcium signals into cellular responses by phosphorylating ion channels and calcium-handling proteins [82]. Oxidative stress promotes AF by triggering CaMKII-mediated phosphorylation of ryanodine receptor 2 (RyR2) at serine 2814, increasing calcium release from the sarcoplasmic reticulum and promoting calcium waves that can cause afterdepolarizations [83]. Additionally, CaMKII phosphorylates Nav1.5 at serine 571, enhancing late sodium currents and contributing to conduction slowing and heterogeneity, further facilitating reentry mechanisms critical to AF pathogenesis [83]. An adenovirus encoding the CaMKII inhibitory peptide CaMKIIn reduced AF susceptibility in a porcine model, significantly improving sinus rhythm maintenance and attenuating structural remodeling. Specifically, CaMKII inhibition decreased atrial fibrosis, apoptosis, and hypertrophy, with cardiomyocyte apoptosis lowered from ~ 1.9% to ~ 0.75%, alongside preserved atrial contractile function [84].

## Clinical Outlook and Future Directions

Despite rapid progress in cardiovascular gene therapy, clinical translation for AF remains in its infancy. The absence of AF-focused trials highlights challenges posed by its multifactorial nature. Recent preclinical work shows promise with AAV9-mediated atrial targeting, RNA-based approaches, and novel delivery systems. Improving atrial-specific targeting remains a top priority. Advances in capsid engineering, non-viral vectors, and atrium-selective promoters may boost precision and safety. Achieving long-term expression without adverse genomic integration is also key. As research continues to clarify AF’s molecular basis, gene therapy is increasingly positioned to prevent or reverse AF at its genetic roots.

## Data Availability

Data sharing not applicable to this article as no datasets were generated or analyzed during the current study.

## Abbreviations

AF: Atrial fibrillation
Ctnt: Cardiac Troponin T
αMHC: Alpha Myosin Heavy Chain
siRNA: Small Interfering RNA
shRNA: Short Hairpin RNA
RNAi: RNA Interference
RISC: RNA-Induced Silencing Complex
miRNA: MicroRNA
ASO: Antisense Oligonucleotide
CRISPR: Clustered Regularly Interspaced Short Palindromic Repeats
LNP: Lipid Nanoparticle
Ad: Adenovirus
AAV: Adeno-Associated Virus
LV: Lentiviral Vector
IV: Intravenous
TGF-β: Transforming Growth Factor-Beta
SOD1: Superoxide Dismutase-1
PI3K: Phosphatidylinositol 3-Kinase
NLRP3: Nucleotide-Binding Domain, Leucine-Rich Repeat, Pyrin Domain-Containing Protein 3
IK: Potassium Current
IK1: Inward Rectifier Potassium Current
IK,Ach: Acetylcholine-Activated Potassium Current
APD: Action Potential Duration
ERP: Effective Refractory Period
INa,L: Late Sodium Current
ICa,L: L-Type Calcium Channel Current
Cx: Connexin
M2R: Muscarinic Type-2 Receptor
ROS: Reactive Oxygen Species
NOX: NADPH Oxidase
RyR2: Ryanodine Receptor 2
CaMKII: Calmodulin Kinase II
